# Effectiveness and Safety of Acupuncture for Migraine: An Overview of Systematic Reviews

**DOI:** 10.1155/2020/3825617

**Published:** 2020-03-23

**Authors:** Yu-Xi Li, Xi-li Xiao, Dong-Ling Zhong, Liao-Jun Luo, Han Yang, Jun Zhou, Ming-Xing He, Li-Hong Shi, Juan Li, Hui Zheng, Rong-Jiang Jin

**Affiliations:** ^1^School of Acupuncture-Moxibustion and Tuina/The Third Affiliated Hospital, Chengdu University of Traditional Chinese Medicine, Sichuan, China; ^2^Hospital of Chengdu University of Traditional Chinese Medicine, Sichuan, China; ^3^School of Health Preservation and Rehabilitation, Chengdu University of Traditional Chinese Medicine, Sichuan, China

## Abstract

**Background:**

Migraine is a common neurological disease, which burdens individuals and society all over the world. Acupuncture, an important method in Traditional Chinese Medicine, is widely used in clinical practice as a treatment for migraine. Several systematic reviews (SRs) have investigated the effectiveness and safety of acupuncture for migraine.

**Objective:**

To summarize and critically assess the quality of relevant SRs and present an objective and comprehensive evidence on the effectiveness and safety of acupuncture for migraine. *Data Sources*. MEDLINE, Embase, Cochrane Library, PROSPERO database, Chinese National Knowledge Infrastructure (CNKI), Chinese Biological Medicine (CBM), China Science and Technology Journal (SCTJ), and WanFang database (WF) were searched from inception to December 2019 and grey literatures were manually searched. *Selection Criteria*. SRs which meet the criteria were independently selected by 2 reviewers according to a predetermined protocol. *Data Extraction*. Characteristics of included SRs were independently extracted by 2 reviewers following a predefined data extraction form. *Review Appraisal*. The methodological quality, risk of bias, and reporting quality of included SRs were assessed, respectively, by a Measurement Tool to Assess Systematic Reviews (AMSTAR) 2, the Risk of Bias in Systematic reviews (ROBIS) tool, and the Preferred Reporting Item for Systematic Review and Meta-analysis-Acupuncture (PRISMA-A) statement. The quality of outcomes was evaluated by the Grading of Recommendations Assessment, Development, and Evaluation (GRADE).

**Results:**

A total of 15 SRs were included. All the SRs were published between 2011–2019. Based on AMSTAR 2, 14 out of 15 SRs were rated critically low quality and 1 was rated low quality. According to ROBIS tool, 9 SRs (60%) were low risk of bias. With the PRISMA-A checklist, we found 11 out of 15 SRs were found adequately reported over 70%. With the GRADE tool, we found high quality of evidence indicated that the effective rate of acupuncture was superior to western medicine in treatment of migraine. Besides, acupuncture reduced more headache days and the times of using painkiller and was more effective in reducing the frequency and degree of headache than western medicine and sham acupuncture. *Limitations*. There might be some missing information. The accuracy of the conclusions may be decreased reduced since we were unable to synthesis all the evidence.

**Conclusions:**

Based on high quality of evidence, we concluded that acupuncture may be an effective and safe therapy for migraine. However, the quality of SRs in acupuncture for migraine still needs more improvement.

## 1. Introduction

Migraine is a common neurological disease characterized by unilateral, throbbing recurrent headache, often accompanied by photophobia, phonophobia, or nausea [1]. According to the epidemiological statistics, the prevalence of migraine is 3.3%∼32.6% in female and 0.7%∼16.1% in male [2]. At the same time, it costs between 6.5 and 17 billion dollars annually in the USA which severely burdened individuals and society [3, 4]. In the Global Burden of Disease Survey 2010, migraine ranked as the 3rd most prevalent disorder and 7th highest specific cause of disability worldwide [5, 6]. The standard treatments for migraine include nonsteroidal anti-inflammatory drugs, antiepileptic drugs, ergotamines, and triptans. However, these pharmacotherapies were often accompanied with undesirable adverse effects [7], such as fatigue, sleep disturbance, nausea, and vomiting [8] which lead to poor compliance of patients. Therefore, more and more patients are seeking for effective nonpharmacological alternative treatments.

Acupuncture, an important method in Traditional Chinese Medicine, is widely used in clinical practice as a treatment for migraine. It is reported that acupuncture was one of the most common complementary therapies in worldwide [9]. In German-speaking countries, acupuncture has high utilization and is one of the most primarily used methods to relieve pain [10]. Now, it is increasingly accepted in western countries as an alternative treatment for migraine and other pain conditions [11]. The results of clinical studies have demonstrated that acupuncture is an effective and safe therapeutic approach to treat migraine [12–14]. With the development of evidence-based medicine, numerous systematic reviews (SRs) have been conducted to investigate the effectiveness and safety of acupuncture for migraine. However, the results of these SRs often have limitations which may lower the quality of conclusions and mislead the patients, clinical doctors, and policy makers.

Overviews of SRs, a method to evaluate the quality of evidence [15], is becoming more and more prevalent in evidence-based medicine [16]. The overview is a comprehensive approach to reassess the quality of SRs by collecting the information of relevant SRs dealing with the same disease or health problem [17]. While SR has always been regarded as one of the most important sources of high quality and reliable information in the evidence-based medicine [18], there are many factors in the evaluation process which can decrease the quality of SRs, such as incomprehensive source of literature, inadequate evaluation method, and publication bias. Overview of SRs comprehensively integrated the evidence of SRs, which contains more information and can provide more high-quality evidence for clinical work.

This is the first overview which comprehensively assessed SRs of acupuncture for migraine with a Measurement Tool to Assess Systematic Reviews (AMSTAR) 2, the Risk of Bias in Systematic reviews (ROBIS), Preferred Reporting Item for Systematic Review and Meta-analysis-Acupuncture (PRISMA-A), and the Grades of Recommendations, Assessment, Development and Evaluation (GRADE). The objective of this overview is to critically assess the quality of relevant SRs and present an objective and comprehensive evaluation on effectiveness and safety of acupuncture for migraine, which can help the public and policy-makers understand whether acupuncture should be recommended as a treatment for migraine.

## 2. Methods

### 2.1. Registration

A predetermined written protocol of this overview was registered in the PROSPERO (International prospective register of systematic overview) database (https://www.crd.york.ac.uk/PROSPERO/), registration number: CRD42017077218. This overview was reported in accordance with the guideline of the pilot version checklist with Preferred Reporting Items for overview of systematic reviews (PRIO-harms) [19].

### 2.2. Ethics

Ethics approval is not required in overview of SRs, since it does not involve individual patient data.

### 2.3. Inclusion Criteria

#### 2.3.1. Types of Reviews

SRs with or without meta-analysis of randomized controlled trials (RCTs) were included in which acupuncture was used as treatment for migraine.

#### 2.3.2. Types of Participants

SRs included RCT recruiting participants diagnosed with migraine according to standard diagnostic criteria (e.g., the International Classification of Headache Disorders released by the International Headache Society or other domestic standards). There was no restriction on the gender, age, race, duration, intensity, condition, and source of the patients.

#### 2.3.3. Types of Interventions

There was no restriction on the types of acupuncture (e.g., body acupuncture, electroacupuncture, auricular acupuncture, warm-acupuncture, and scalp acupuncture).

#### 2.3.4. Types of Comparators

SRs included control groups which were treated with sham-acupuncture, placebo, medicine, and other types of nonpharmaceutical therapy or placed in the waiting list.

#### 2.3.5. Types of Outcomes

The primary outcome was effective rate. Secondary outcomes included intensity, frequency or duration of headache, times of using painkiller, quality of life, recurrent rate, and adverse effects of acupuncture in migraine.

### 2.4. Exclusion Criteria

The SRs were excluded if one of the following criteria was met: did not use the diagnostic criteria of migraine mentioned above; SRs with network meta-analysis or indirect comparison; SRs that included retrospective studies, prospective studies, cross-sectional clinical studies, and case reports; SRs whose data could not be extracted; duplicated publication; review comments.

### 2.5. Search Strategy

An electronic literature search was conducted in the MEDLINE, Embase, Cochrane Library, PROSPERO database, Chinese National Knowledge Infrastructure (CNKI), Chinese Biological Medicine (CBM) database, Chinese Science and Technology Periodical Database (SCTJ), and WanFang database, all from the inception to December 2019. Details of search strategy were presented in Supplementary [Supplementary-material supplementary-material-1]. In addition, reference lists/bibliographies of included studies, study registries, and grey literature, such as dissertations and conference reports, were also searched to avoid missing studies. Besides, the experts in the field were also consulted. No language restrictions were applied.

### 2.6. Screening

The reviewer (JZ) searched the databases according to the predeveloped standardized search strategy. All the retrieved literatures were imported into Endnote X8. Two reviewers (HY and YXL) independently screened for candidates according to the inclusion and exclusion criteria by reading the title and abstract. Then, the full texts were downloaded for further screening. At the same time, bibliographic references were also reviewed to identify possible SRs. The disagreements were resolved by discussion. If necessary, the discrepancies were resolved by consulting the third reviewer (DLZ).

### 2.7. Data Extraction

A data extracted form was predefined, including the characteristics of SRs, such as author, title, published year, sample size, intervention, outcome indicators, quality evaluation method, and conclusion. Data was independently extracted by two reviewers (HY and LJL) using Microsoft Excel. After extraction, the two reviewers (LHS and YXL) cross checked to eliminate mis-entry. Discrepancies were resolved by team discussion or arbitrated by the third reviewer (DLZ).

### 2.8. Assessment of SRs

The assessment of included SRs was carried out independently by qualified reviewers who were trained in the Chinese Cochrane Center. Before the evaluation, each topic of the assessment tools was intensively discussed to achieve consensus. After evaluation, two reviewers cross checked the results. Discrepancies were resolved by team discussion or an independent decision form a third reviewer.

1. AMSTAR 2 [20] was used to assess the methodological quality of included SRs. The checklist has 16 items, including 7 critical items (items 2, 4, 7, 9, 11, 13, and 15), which are used to critically assess the validity of an SR. Each item was evaluated as “yes” (a positive result), “partial yes” (partial adherence to the standard), and “no” (no information is provided to rate an item) according to adherence to the standard.
2. The aim of the ROBIS tool is to evaluate the level of bias presented in a systematic review. This tool assesses the level of bias across 4 domains of 2 phases: “study eligibility criteria,” “identification and selection of studies,” “data collection and study appraisal,” and “synthesis and findings”. Each domain has signaling questions and a judgment of concerns about risk of bias of the domain, and the results are rated as “high risk,” “low risk,” or “unclear risk” [21].
3. PRISMA-A statements an extension of PRISMA especially for acupuncture, which was published in 2019 [22]. It consists of a 27-item checklist and a 4-phase flow diagram, aiming to help authors improve the reporting quality of SRs on acupuncture interventions. Seven aspects of SRs include title, abstract, introduction, methods, results, discussion and funding. Response options for each item are “yes,” “no,” and “not applicable”. The completion of each item was presented as a ratio.
4. The quality of primary outcomes of included SRs was evaluated by the GRADE system [23]. The assessment of included SRs was carried out independently by qualified reviewers (JL and DLZ) who were trained in the GRADE Center in China (Lanzhou). The 5 key elements of GRADE influenced the quality of evidence including study limitations, inconsistency of results, indirectness of evidence, imprecision, and reporting bias. The quality of evidences of SRs was rated as “High,” “Moderate,” “Low,” and “Very Low”. Evidence based on RCTs began as high quality.

## 3. Results

### 3.1. Literature Search

**Figure 1:**
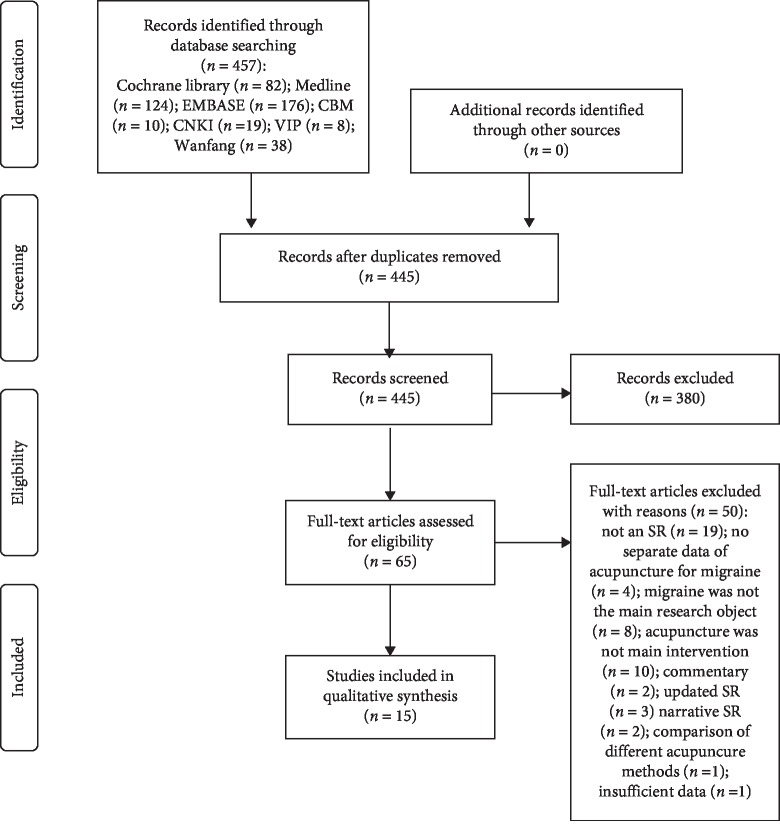
Flowchart of the selection process of included SRs.

We retrieved 457 records according to the search strategy. 11 duplicates were excluded by filtration, 445 papers were screened by titles and abstracts. 65 articles were considered eligible, and full-text papers were downloaded. After being reviewed by two reviewers independently, 50 SRs were excluded and 15 SRs [24–38] were included for further analyses (Figure 1). The reasons for exclusion are presented in Supplementary [Supplementary-material supplementary-material-1].

### 3.2. Characteristics of SRs

The characteristics of included SRs are presented in Table 1. All the included SRs were published between 2011–2019, 6 of which were published in 2016 [21, 31–34, 36]. The number of RCTs in SRs ranged from 2 to 33. 3 SRs were on prophylactic treatment for migraine [31, 33, 35], 1 SR on acute migraine [33], 1 SR for menstrual migraine [36], 1 SR for migraine without aura [37], and the others did not clearly stated the type of migraine. 11 SRs specified the diagnostic criteria of HIS (International Headache Society) or ICHD (International Classification of Headache Disorders), while 4 SRs [25, 27, 28, 32] did not report the diagnostic criteria. All the 15 SRs performed meta-analysis, 10 out of 15 SRs [24–29, 31, 32, 35, 38] performed subgroup analysis, and only 4 SRs [30, 33, 34, 36, 38] conducted sensitivity analysis. The intervention was acupuncture, while comparators were mainly sham acupuncture and medications (ergotamine, ibuprofen, flunarizine, nimodipine, celecoxib, aspirin, somedon, sodium valproate, metoprolol, and topiramate). The outcomes of SRs were effective rate, intensity, and frequency or duration of headache. For the assessment of methodological quality, 2 SRs [24, 29] used the Jadad scale, 12 SRs [25, 26, 30–34, 36, 38] used the Cochrane risk of bias tool, and the remaining 1 SR [27] did not report any specific tool but described 6 aspects of quality assessment, including randomization, allocation concealment, blind method, data integrity, selective reporting, and other biases.

### 3.3. Methodological Quality of Included SRs

An overview of methodological quality of included SRs is presented in Table 2. Among the 15 SRs, 14 were rated critically low quality and 1 was rated low quality [37]. Items 2, 3, 7, 10, and 16 were rated particularly low quality. All SRs used satisfactory techniques to assess the risk of bias. Only 1 SR [37] established a prior study protocol and 2 [28, 36] reported the funding sources of the included studies. No SR explained the reasons for selection of study types or provided a complete list of excluded studies with reasons. And, few SRs assessed publication bias by a funnel plot.

### 3.4. Risk of Bias of Included SRs

**Figure 2:**
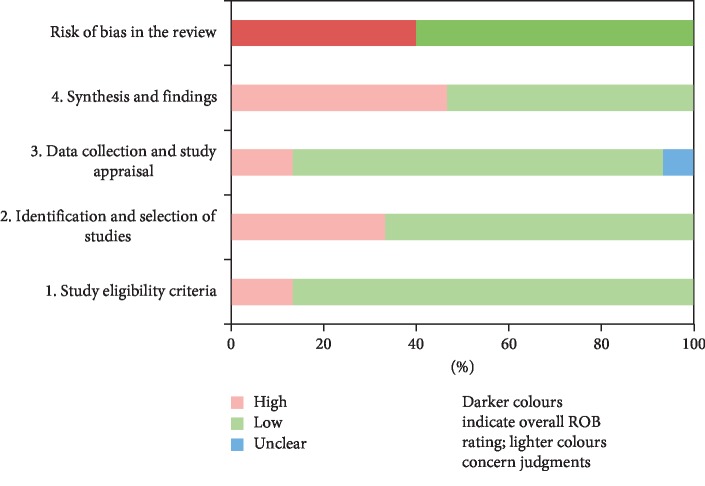
Graphical presentation of risk of bias of included SRs.

The ROBIS tool, containing 3 phases with 4 domains, was used to assess the risk of bias of included SRs. Phase 1 assesses the relevance of research question, which is optional and was not performed in our study.  Table 3 and Figure 2 present the assessment of risk of bias of each SR. Domain 1 assessed concerns regarding specification of study eligibility criteria, and 12 of 15 SRs (80%) were rated low risk of bias. Domain 2 assessed concerns regarding methods used to identify and select studies, in which 9 SRs (60%) were in low risk of bias. Domain 3 assessed concerns regarding methods used to collect data and appraise studies, and 11 SRs (73%) were at low risk of bias and 1 [24] unclear risk of bias. Domain 4 assessed concerns regarding the synthesis and findings, and 8 SRs (53%) were rated as low risk of bias. The final phase considered the overall risk of bias of SRs, and 9 SRs (60%) were low risk of bias.

### 3.5. Reporting Quality of Included SRs

Table 4 presents the overview of PRISMA-A checklist items. 11 out of 15 SRs were adequately reported over 70%. The section of title, abstract, and introduction were all well reported (100%). Though in Section 2, topic of protocol and registration, search strategy, study selection, data items, risk of bias, and additional analyses were reported inadequately, three topics (study selection, risk of bias across studies, and additional analysis) in Section 3 were reported under 70%. Of all the items, protocol and registration (13.33%), search strategy (33.33%), risk of bias in individual studies (33.33%), and risk of bias across studies (33.33%) accounted for the main reporting limitations. Overall, 4 SRs [26, 36–38] reached over 85% compliance.

### 3.6. Effectiveness of Acupuncture for Migraine

We summarized the outcomes from the included SRs and presented them in Table 5. The evidence suggested that the effective rate of acupuncture was superior to western medicine (risk ratio (RR) = 1.17, 95% confidence interval (CI) = (1.12, 1.22), *P* = 0.71) [26]. Acupuncture had better long-term effective rate for migraine (RR = 4.17, 95% CI (2.80, 6. 20), *P* < 0.00001) [34, 35] and reduced more headache days (standardized mean difference (SMD) = −0.13, 95% CI = (−0.25, −0.02), *P* = 0.02) and the times of using painkiller (SMD = −0.73, 95% CI = (−2.14, 0.69), *P* = 0.31) than western medicine and sham acupuncture, both in short-term and long-term follow-up [33, 35]. Besides, acupuncture was more effective in reducing frequency (SMD = −2.18, 95% CI = (−2.61, −1.75), *P* < 0.00001) and degree of headache (SMD = −1.93, 95% CI = (−2.53, −1.36), *P* = 0.005) than western medicine and sham acupuncture [31, 35]. One SR [25] reported more effective rate of acupuncture than Chinese herbal medicine in treating migraine (RR = 1.29, 95% CI = (1.14, 1.45), *P* < 0.00001).

### 3.7. Evidence Quality of Included SRs

We evaluated the quality of primary outcomes extracted from included studies.  Table 6 shows the level of evidence quality of studies reported effective rate. The high level of evidence quality indicated that the effective rate of acupuncture was superior than western medicine, both in short-term and long-term. The inconsistency and imprecision were the main reasons for downgrading. Significant heterogeneity downgraded inconsistency and imprecision was downgraded because the total sample size did not meet the optimal information size.

### 3.8. Safety of Acupuncture for Migraine

Of all the 15 SRs, 8 SRs [26, 30, 31, 33–37] mentioned the adverse events of acupuncture in the treatment of migraine. 2 SRs [30, 36] did not further analyze the safety evaluation due to the small number of studies. 6 SRs [26, 31, 33–35, 37] concluded that acupuncture treatment had fewer adverse events than medication, which indicated that acupuncture was a safe therapy for migraine.

## 4. Discussion

### 4.1. Summary of Main Findings

This is the first overview of SRs that investigate the effectiveness and safety of acupuncture for migraine. We rigorously appraised the published SRs with AMSTAR 2, ROBIS, PRISMA-A, and GRADE. Based on AMSTAR 2, 14 out of 15 SRs were rated critically low quality and 1 was rated low quality. By using the ROBIS tool, 9 SRs were rated low risk bias. With PRISMA-A checklist, we found 11 out of 15 SRs were found adequately reported over 70%. The results of GRADE suggested that acupuncture was is an effective and safe method for migraine.

### 4.2. Implications for Further Study

This overview presents several challenges for producers of SRs that should be considered. By using the ROBIS tool, we found that the risk of bias in domain 2 and domain 4 of phase 2 were relatively high. In domain 2, we focused on the risk of bias in identification and selection of studies. The results indicated that the reviewers of SR should pay attention to whether the search includes an appropriate range of databases or electronic sources for published reports. Instead of database searching, the additional methods should also be used to identify relevant reports, including conference reports and clinical trial registration platforms. In domain 4, the risk of bias in synthesis of findings was high. Even though the data was synthesized in all the SRs, we were not able to determine whether data synthesis and analysis methods have been followed in advance, which may ignore the results of some studies. The robustness of the findings should be assessed through funnel plot or sensitivity analyses, and the biases in primary studies should be minimized or addressed in the synthesis.

The PRISMA-A statement provided the basis for the author to improve the reporting quality of the SRs with acupuncture as intervention. According to the results of the PRISMA score, the lowest report rate (13.33%) was in the protocol and registration section. Only 2 SRs managed to offer a protocol or registration number of SR. An advance registration helps promote transparency, minimize potential bias in the conducting and reporting review, reduce duplication of effort between groups, and keep SRs updated. [39] A free and open database, the International Prospective Register of Systematic Reviews (PROSPERO, http://www.crd.york.ac.uk/prospero), has been advocated and recommended for reviewers to avoid bringing bias in SRs. In order to achieve a better quality of evidence, the researchers need to strictly control the risk of bias with reference to the ROBIS tool when conducting SRs/meta-analyses. In accordance with the requirements of the PRISMA-A statement, writing a SR/meta-analysis helps to get better reporting quality.

In the assessment of evidence quality with GRADE tool, we found that the biggest reason for downgrading was inconsistencies among studies, owing to the high I^2^ value and statistically significant heterogeneity of effect estimates. The GRADE guideline suggests, when it comes to inconsistency, SR authors should generate and test a small number of a priori hypotheses related to patients, interventions, outcomes, and methodology to explore the sources of heterogeneity [40].

### 4.3. Strength and Limitations

There are some strength in our study; firstly, this overview is the first to systematically evaluated the methodological quality and reporting quality of SRs in acupuncture for migraine. Secondly, we combined the latest high-quality evidence of SRs to provide a more convinced evidence for clinical work. Thirdly, we started this overview with a predesigned protocol, which helped reduce the risk of bias.

In addition to the strengths, there are several limitations to be noted. Firstly, there might be some missing information since we only gathered studies in English and Chinese. Secondly, we were unable to synthesize all the evidence, which may decrease the accuracy of the conclusions.

## 5. Conclusions

Based on high quality of evidence, we conclude that acupuncture is more effective and safer than medication or sham acupuncture in the treatment of migraine. However, the methodological quality, risk of bias, and reporting quality of SRs in acupuncture for migraine still needs improvement in future.

## Figures and Tables

**Table 1:** Characteristics of included SRs.

First author and year of publication	Included study design	No. of study	No. of patient	Diagnostic criteria	Intervention	Comparator	Outcomes	Assessment of methodological quality	Meta-analysis conducted?	Subgroup analysis conducted?	Sensitivity analysis conducted?	Safety
Gao (2011)	RCT	12	1744	IHS; ICHD-10	Acupuncture	Sham acupuncture	Effective rate; headache days	Jadad scale	Yes	Yes	No	NR
Zheng (2012)	RCT	33	3593	NR	Acupuncture	Herbs; sham acupuncture	Effective rate	Cochrane risk of bias tool	Yes	Yes	No	NR
Chen (2014)	RCT	18	1672	IHS	Acupuncture	Medications (flunarizine, ibuprofen, nimodipine, naproxen, ergotamine caffeine); herbs	Effective rate; headache frequency; headache intensity and duration; accompanying symptoms	Cochrane risk of bias tool	Yes	Yes	Yes	Yes
Yang (2014)	RCT	10	893	NR	Acupuncture	Flunarizine	Short-term effect; long-term effect; headache score	NR	Yes	No	No	Yes
Zhao (2014)	RCT	17	1719	NR	EA	Medications	Effective rate; VAS	Cochrane risk of bias tool	Yes	Yes	No	Yes
Dai (2015)	RCT	2	140	IHS	Acupuncture	Medications (flunarizine, nimodipine)	Cure rate; effective rate	Jadad scale	Yes	No	No	NR
Yang (2015)	RCT	10	997	IHS	Acupuncture	Sham acupuncture	Effective rate; headache intensity; headache frequency; headache duration; medication use; accompanying symptoms	Cochrane risk of bias tool	Yes	Yes	No	Yes
Linde (2016)	RCT	22	4985	IHS	Acupuncture	Sham interventions; medications (metoprolol, flunarizine, valproic acid); waiting list	Headache frequency; the proportion of “responders”	Cochrane risk of bias tool	Yes	Yes	No	NR
Pu J (2016)	RCT	5	618	NR	Acupuncture	Sham acupuncture	VAS	Cochrane risk of bias tool	Yes	No	No	No
Pu (2016)	RCT	7	1285	IHS	Acupuncture	Medications (flunarizine, valproate, topiramate, metoprolol)	Effective rate; headache frequency; headache intensity; medication use	Cochrane risk of bias tool	Yes	No	Yes	Yes
Song (2016)	RCT	18	1470	IHS; ICHD	Acupuncture; EA	Medications (flunarizine, ibuprofen, nimodipine, metoprolol); placebo	Long-term effective rate; short-term effective rate; headache days	Cochrane risk of bias tool	Yes	Yes	Yes	Yes
Xian (2016)	RCT	26	3657	IHS; ICHD	Acupuncture	Sham acupuncture; medications (flunarizine, sodium valproate, metoprolol, topiramate, nimodipine)	Effective rate; headache frequency; VAS; medication use; PDI; MIDAS; PF-SF36; MH-SF36	Cochrane risk of bias tool		Yes	No	Yes
Zhao (2016)	RCT	18	1268	IHS	Acupuncture; auricular acupuncture; EA	Medications (celecoxib, flunarizine, aspirin, ibuprofen, somedon); herbs	Effective rate; VAS; headache intensity; headache frequency	Cochrane risk of bias tool		Yes	Yes	Yes
Xu (2018)	RCT	14	1155	ICHD	Acupuncture; EA	Medications (ergotamine, ibuprofen, flunarizine, nimodipine); sham acupuncture	Frequency of migraine attacks; number of migraine days; VAS; effective rate	Cochrane risk of bias tool	Yes	No	No	Yes
Lu (2019)	RCT	17	2226	ICHD-3	Acupuncture; EA	Sham acupuncture; medications (ergotamine, flunarizine, nimodipine)	Frequency of migraine attacks; duration of migraine; headache intensity	Cochrane risk of bias tool	Yes	Yes	Yes	NR

IHS = International Headache Society; ICHD = International Classification of Headache Disorders; NR = no report; AA = auricular acupuncture; EA = electro-acupuncture; acupuncture = classical manual acupuncture; VAS = Visual Analogue Scale/Score; PDI = Pain Disability Index; MIDAS = the Migraine Disability Assessment; SF-36/12 = Short Form 36/12 Questionnaire.

**Table 2:** Methodological quality of included SRs on acupuncture for migraine.

Author (year)	I1	I2^*∗*^	I3	I4^*∗*^	I5	I6	I7^*∗*^	I8	I9^*∗*^	I10	I11^*∗*^	I12	I13^*∗*^	I14	I15^*∗*^	I16	Ranking of quality
Gao (2011)	Y	N	N	PY	N	N	N	Y	Y	N	N	Y	Y	N	Y	N	Critically low
Zheng (2012)	Y	N	N	PY	Y	Y	N	N	Y	N	N	Y	Y	N	N	N	Critically low
Chen (2014)	Y	N	N	PY	Y	Y	N	PY	Y	N	Y	Y	Y	N	Y	N	Critically low
Yang (2014)	Y	N	N	PY	Y	N	N	PY	Y	N	N	N	N	N	N	N	Critically low
Zhao (2014)	Y	N	N	PY	Y	Y	N	PY	Y	Y	Y	N	N	N	N	N	Critically low
Dai (2015)	N	N	N	PY	N	N	N	N	Y	N	N	N	N	Y	N	N	Critically low
Yang (2015)	Y	N	N	PY	N	Y	N	PY	Y	N	Y	Y	Y	Y	N	N	Critically low
Linde (2016)	Y	N	N	PY	Y	Y	N	PY	Y	N	Y	Y	Y	N	N	N	Critically low
PuJ (2016)	Y	N	N	PY	Y	Y	N	N	Y	N	Y	N	N	Y	N	N	Critically low
Pu (2016)	Y	N	N	PY	Y	Y	N	Y	Y	N	Y	Y	N	Y	N	N	Critically low
Song (2016)	Y	N	N	N	Y	Y	N	PY	Y	N	Y	Y	N	Y	Y	N	Critically low
Xian (2016)	Y	N	N	PY	Y	Y	N	Y	Y	N	Y	N	N	Y	N	N	Critically low
Zhao (2016)	Y	N	N	PY	Y	Y	N	Y	Y	Y	Y	Y	Y	N	Y	N	Critically low
Xu (2018)	Y	Y	N	PY	Y	Y	N	Y	Y	N	Y	N	Y	Y	Y	Y	Low
Lu (2019)	Y	N	N	PY	Y	Y	N	Y	Y	N	Y	N	Y	Y	Y	N	Critically low

^*∗*^The key items of the AMSTAR 2; I: item; Y: yes; N: no; PY: partial yes. Item 1: did the research questions and inclusion criteria for the review include the components of PICO? Item 2: did the report of the review contain an explicit statement that the review methods were established prior to the conduct of the review and did the report justify any significant deviations from the protocol? Item 3: did the review authors explain their selection of the study designs for inclusion in the review? Item 4: did the review authors use a comprehensive literature search strategy? Item 5: did the review authors perform study selection in duplicate? Item 6: did the review authors perform data extraction in duplicate? Item 7: did the review authors provide a list of excluded studies and justify the exclusions? Item 8: did the review authors describe the included studies in adequate detail? Item 9: did the review authors use a satisfactory technique for assessing the risk of bias (RoB) in individual studies that were included in the review? Item 10: did the review authors report on the sources of funding for the studies included in the review? Item 11: if meta-analysis was performed did the review authors use appropriate methods for statistical combination of results? Item 12: if meta-analysis was performed, did the review authors assess the potential impact of RoB in individual studies on the results of the meta-analysis or other evidence synthesis? Item 13: did the review authors account for RoB in individual studies when interpreting/discussing the results of the review? Item 14: did the review authors provide a satisfactory explanation for, and discussion of, any heterogeneity observed in the results of the review? Item 15: if they performed quantitative synthesis, did the review authors carry out an adequate investigation of publication bias (small study bias) and discuss its likely impact on the results of the review? Item 16: did the review authors report any potential sources of conflicts of interest, including any funding they received for conducting the review?

**Table 3:** Tabular presentation of risk of bias of included SRs.

Review	Phase 2			Phase 3	
	1. Study eligibility criteria	2. Identification and selection of studies	3. Data collection and study appraisal	4. Synthesis and findings	Risk of bias in the review
Gao (2011)	☺	☹	?	☺	☹
Zheng (2012)	☺	☺	☺	☹	☹
Chen (2014)	☺	☺	☺	☺	☺
Yang (2014)	☺	☹	☹	☹	☹
Zhao (2014)	☹	☹	☺	☺	☹
Dai (2015)	☺	☹	☹	☹	☹
Yang (2015)	☺	☺	☺	☺	☺
Linde (2016)	☺	☺	☺	☺	☺
PuJ (2016)	☹	☹	☺	☹	☹
Pu (2016)	☺	☺	☺	☺	☺
Song (2016)	☺	☺	☺	☺	☺
Xian (2016)	☺	☺	☺	☹	☺
Zhao (2016)	☺	☺	☺	☺	☺
Xu (2018)	☺	☺	☺	☹	☺
Lu (2019)	☺	☺	☺	☹	☺

☺ = low risk; ☹ = high risk; ? = unclear risk.

**Table 4:** Compliance of included SRs with PRISMA-A checklist.

	Section/topic		Gao (2011)	Zheng (2012)	Chen (2014)	Yang (2014)	Zhao (2014)	Dai (2015)	Yang (2015)	Linde (2016)	PuJ (2016)	Pu (2016)	Song (2016)	Xian (2016)	Zhao (2016)	Xu (2018)	Lu (2019)	Compliance (%)
1	Title	Title	Y	Y	Y	Y	Y	Y	Y	Y	Y	Y	Y	Y	Y	Y	Y	100.00
2	Abstract	Structured summary	Y	Y	Y	Y	Y	Y	Y	Y	Y	Y	Y	Y	Y	Y	Y	100.00
3	Introduction	Rationale	Y	Y	Y	Y	Y	Y	Y	Y	Y	Y	Y	Y	Y	Y	Y	100.00
4		Objectives	Y	Y	Y	Y	Y	Y	Y	Y	Y	Y	Y	Y	Y	Y	Y	100.00
5	Methods	Protocol and registration	N	N	N	N	N	N	N	Y	N	N	N	N	N	Y	N	13.33
6		Eligibility criteria	Y	N	Y	Y	Y	N	Y	Y	Y	Y	Y	Y	Y	Y	Y	86.67
7		Information sources	Y	Y	Y	N	Y	Y	Y	N	N	Y	Y	Y	Y	Y	Y	80.00
8		Search	N	N	Y	N	N	N	Y	Y	N	N	N	N	Y	N	Y	33.33
9		Study selection	Y	N	Y	Y	N	N	N	Y	N	Y	N	Y	N	Y	Y	53.33
10		Data collection process	N	Y	Y	Y	Y	N	Y	Y	Y	Y	Y	N	Y	Y	Y	80.00
11		Data items	N	N	Y	Y	N	N	Y	Y	N	Y	N	Y	Y	Y	Y	60
12		Risk of bias in individual studies	Y	Y	Y	Y	Y	N	Y	Y	Y	Y	Y	Y	Y	Y	Y	93.33
13		Summary measures	Y	Y	Y	Y	N	Y	Y	Y	Y	Y	Y	Y	Y	Y	Y	93.33
14		Synthesis of results	Y	Y	Y	Y	Y	Y	Y	Y	Y	Y	Y	Y	Y	Y	Y	100.00
15		Risk of bias across studies	Y	N	Y	N	N	N	N	N	N	N	Y	N	Y	Y	N	33.33
16		Additional analyses	Y	Y	Y	N	N	N	Y	Y	N	Y	Y	Y	Y	Y	Y	73.33
17	Results	Study selection	Y	N	Y	Y	N	N	N	Y	N	Y	Y	N	Y	N	N	53.33
18		Study characteristics	Y	N	Y	Y	Y	N	Y	Y	Y	Y	Y	Y	Y	Y	Y	86.67
19		Risk of bias within studies	Y	Y	Y	Y	Y	N	Y	Y	Y	Y	Y	Y	Y	Y	Y	93.33
20		Results of individual studies	Y	Y	Y	Y	Y	Y	Y	Y	Y	Y	Y	Y	Y	Y	Y	100.00
21		Synthesis of results	Y	Y	Y	Y	Y	Y	Y	Y	Y	Y	Y	Y	Y	Y	Y	100.00
22		Risk of bias across studies	Y	N	Y	N	N	N	N	N	N	N	Y	N	Y	Y	N	33.33
23		Additional analysis	Y	N	Y	N	N	N	Y	Y	N	Y	N	N	Y	N	Y	46.67
24	Discussion	Summary of evidence	Y	Y	Y	Y	Y	Y	Y	Y	Y	Y	Y	Y	Y	Y	Y	100.00
25		Limitations	N	Y	Y	Y	Y	N	Y	Y	N	Y	Y	Y	Y	Y	Y	86.67
26		Conclusions	Y	Y	Y	Y	Y	N	Y	Y	Y	Y	Y	Y	Y	Y	Y	93.33
27	Funding	Funding	N	N	N	Y	N	N	N	Y	Y	N	Y	N	Y	Y	Y	46.67
			77.78%	59.26%	92.59%	74.07%	59.26%	37.03%	77.78%	88.89%	59.26%	81.48%	81.48%	70.37%	92.59%	88.89%	88.89%	

**Table 5:** Summary of evidence.

Author (year)	Outcomes (total patient number in the intervention group/total patient number in the control group or total participants in both groups, number of studies)
Acupuncture vs sham-acupuncture	
Gao (2011)	Effective rate (OR = 1.28, 95% C (1.02, 1.61), *P*=0.03) (650/603, 8)
Zheng (2012)	Effective rate (RR = 1.87, 95% CI (1.17, 2.98), *P*=0.009) (91/54, 3)
Chen (2014)	Effective rate (RR = 1.19, 95% CI (1.13, 1.25), *P*=0.06), (596/438, 13); headache times (SMD = 0.75, 95% CI (0.42, 1.08), *P*=0.001), (362/288, 7); headache degree (SMD = 0.47, 95% CI (−0.17, 1.10), *P* < 0.00001) (330/258, 6); headache duration (SMD = 0.62, 95% CI (0.46, 0.78), *P*=0.008), (362/288, 7)
Yang (2014)	Short-term effective rate (RR = 1.27, 95% CI (1.11, 1.45), *P* < 0.0004), (414/409, 9); long-term effective rate (RR = 1.76, 95% CI (1.05, 2.94), *P*=0.03), (117/115, 4)
Zhao (2014)	Effective rate (RR = 1.18, 95% CI (1.09, 1.27), *P*=0.007) (619/410, 11)
Dai (2015)	Effective rate (OR = 4.85, 95% CI (1.69, 13.94), *P*=0.003), (65/51, 2)
Yang (2015)	Not effective rate (RR = 0.24, 95% CI (0.15, 0.38), *P*=0.61), (19/93, 4); recurrence rate (RR = 0.47, 95% CI (0.28, 0.81), *P*=93), (14/53, 2)
Linde (2016)	Headache frequency after treatment (SMD = −0.18, 95% CI (−0.28, −0.08), I^2^ = 47%), (952/694, 12); headache frequency after follow-up (SMD = −0.18, 95% CI (−0.28, −0.08), I^2^ = 47%), (896/638, 10)
PuJ (2016)	VAS score 2 h after acupuncture (MD = −0.38, 95% CI (−0.83, 0.07), *P*=0.10), (350/349, 4); reduced VAS score 2 h after acupuncture (MD = 0.36, 95% CI (0.08, 0.65), *P*=0.01), (290/289, 3); VAS score 4h after acupuncture (MD = −0.42, 95% CI (−0 .96, 0.12), *P*=0.12), (350/349, 4); reduced VAS score 4h after acupuncture (MD = 0.49, 95% CI (0.14, 0.84), *P*=0.007), (290/289, 3)
Xian (2016)	Effective rate at 1–2 months follow-up (RR = 1.06, 95% CI (0.92, 1.24), *P*=0.42), (508/462, 5); effective rate at 3–4 months follow-up (RR = 1.06, 95% CI (0.91, 1.22), *P*=0.48), (525/476, 6); effective rate at 5–6 months follow-up (RR = 1.11, 95% CI (0.96, 1.29), *P*=0.17), (515/470, 5); effective rate of more than 6 months follow-up (RR = 2.03, 95% CI (1.10, 3.74), *P*=0.02), (24/11, 2)
Xu (2018)	Headache frequency (MD = 1.05, 95% CI (1.75, 0.34); *P* < 0.01), (120/120, 3); VAS score (MD = 1.19, 95% CI (1.75, 0.63); *P* < 0.01), (84/84, 3)
Lu (2019)	Headache frequency (SMD = −0.97, 95% CI (−1.60,−0.34), *P*=0.002), (95/69, 3); headache duration (SMD = −0.73, 95% CI (−1.25,−0.21),*P*=0.006) (86/82, 3); headache intensity (SMD = −0.67, 95% CI (−1.15, −0.19),*P*=0.006), (553/490, 6)
Acupuncture vs western medicine	
Zheng (2012)	Effective rate (RR = 1.24, 95% CI (1.16, 1.34), *P* < 0.00001), (1602/925, 28)
Linde (2016)	Headache frequency after treatment (SMD = −0.25, 95% CI (−0.39, −0.10)), (431/308, 3); headache frequency after follow-up (SMD = −0.13, 95% CI (−0.28, −0.01)), (436/308, 3)
Pu (2016)	Effective rate after 3–4 months(RR = 1.24, 95% CI (1.04, 1.47), *P*=0.02), (449/323, 4); effective rate after 5–6 months (RR = 1.18, CI (0.97, 1.43), *P*=0.11), (344/220, 2); headache days after 3–4 months (SMD = -0.30, 95% CI (−0.45,−0.16), *P* < 0.0001), (439/316, 4); headache days after 5–6 months (MD = −0.66, 95% CI (−1.18,−0.13), *P*=0.01), (344/220, 2); headache times after 3–4 months (MD = −0.32, 95% CI (−0.59,-0.04), *P*=0.03), (171/145, 3); headache times after 3–4 months (MD = −0.47, 95% CI (−1.22,−0.28), *P*=0.22), (131/106, 2); headache degree after 3–4 months (SMD = −0.11, 95% CI (−0.56, 0.33), *P*=0.01), (495/370, 4); headache degree after 5–6 months (SMD = −0.31, 95% CI (−0.47, −0.15), *P*=0.0001), (385/261, 3); Times of using painkiller after 3–4 months(MD = −0.64, 95% CI (−1.93, 0.65), *P*=0.33), (207/181, 4); times of using painkiller after 5–6 months(SMD = −0.22, 95% CI (−0.44, 0.00), *P*=0.06), (174/147, 3)
Song (2016)	Short-term effective rate (RR = 2.76, 95% CI (2.03, 3.77), *P* < 0.00001), (616/602, 15); long-term effective rate(RR = 4.17, 95% CI (2.80, 6.20), *P* < 0.00001), (331/311, 7); headache times (RR = −0.79, 95% CI (−1.39, −0.20), *P*=0.009), (92/72, 2)
Xian (2016)	Effective rate at 0–1 months follow-up (RR = 1.66, 95% CI (1.16, 2.37), *P*=0.005), (180/160, 4); effective rate at 1–2 months follow-up (RR = 1.25, 95% CI (1.01, 1.55), *P*=0.04), (162/76, 2); effective rate at 3–4 months follow-up (RR = 1.55, 95% CI (1.09, 2.20), *P*=0.01), (239/125, 5); effective rate at 5–6 months follow-up (RR = 1.30, 95% CI (0.77, 2.19), *P*=0.32), (169/87, 2)
Zhao (2016)	Effective rate (RR = 1.18, 95% CI (1.09, 1.27), *P*=0.007), (649/497, 11);
Xu (2018)	Headache frequency (MD = 1.50; 95% CI (2.32, 0.68); *P* < 0.01), (110/110, 2); VAS score (MD = 0.97, 95% CI (0.63, 1.31); *P* < 0.01), (198/163, 3); effective rate (RR = 1.30; 95% CI (1.16, 1.45); *P* < 0.01), (178/178, 6)
Lu (2019)	Headache frequency (SMD = −1.29, 95% CI (−1.85,−0.73), *P* < 0.00001), (512/486, 8); headache duration (SMD = −0.88, 95% CI (−1.32, −0.45), *P* < 0.0001) (445/427, 7)
Acupuncture vs Chinese herbal medicine	
Zheng (2012)	Effective rate (RR = 1.29, 95% CI (1.14, 1.45), *P* < 0.00001), (111/81, 3)

CI, confidence interval; OR, odds ratio; RR, relative risk; MD, mean difference; WMD, weighted mean difference; SMD, standardized mean difference, HR, hazard ratio; VAS, visual analogue scale.

**Table 6:** Evidence quality of included studies.

Author (date)	Interventions vs comparisons	Outcomes (number of studies)	Risk of bias	Inconsistency	Indirection	Imprecision	Publication bias	Quality of evidence
Gao (2011)	Acupuncture vs sham acupuncture	Effective rate at the end of treatment (8)	0	0	0	−1^①^	0	Moderate
		Effective rate at the end of follow-up (4)	0	−1^②^	0	−1^①^	0	Low
Zheng (2012)	Acupuncture vs western medicine	Effective rate (8)	0	−1^②^	0	0	0	Low
	Acupuncture vs Chinese medicine therapy	Effective rate (3)	0	0	0	−1^①^	−1^③^	Low
	Acupuncture vs sham acupuncture	Effective rate (3)	0	0	0	−1^①^	−1^③^	Low
Chen (2014)	Acupuncture vs western medicine	Effective rate (13)	0	0	0	0	0	High
Yang (2014)	Acupuncture vs western medicine	Short-term effective rate (9)	0	−1^②^	0	0	0	Low
		Long-term effective rate (4)	0	−1^②^	0	—1^①^	0	Very low
Zhao (2014)	Acupuncture vs Western medicine	Effective rate (11)	0	0	0	0	0	High
Dai (2014)	Acupuncture vs western medicine	Effective rate (2)	0	−1^②^	0	−1^①^	−1^③^	Very low
Pu (2016)	Acupuncture vs western medicine	Effective rate after 3–4 months follow-up (4)	0	−1^②^	0	−1^①^	−1^③^	Very low
		Effective rate after 5–6 months follow-up (2)	0	0	0	−1^①^	−1^③^	Low
Song (2016)	Acupuncture vs western medicine	Short-term effective rate (15)	0	0	0	0	0	High
		Long-term effective rate (7)	0	0	0	0	0	High
		Long-term headache times (2)	0	−1^②^	0	0	−1^③^	Low
Xian (2016)	Acupuncture vs sham acupuncture	Effective rate at 1–2 months follow-up (5)	0	0	0	−1^①^	0	Moderate
		Effective rate at 3–4 months follow-up (6)	0	0	0	−1^①^	0	Moderate
		Effective rate at 5–6 months follow-up (5)	0	−1^②^	0	−1^①^	0	Low
		Effective rate of more than 6 months follow-up (2)	0	−1^②^	0	0	−1^③^	Low
	Acupuncture vs western medicine	Effective rate at 1–2 months follow-up (4)	0	−1^②^	0	0	−1^③^	Low
		Effective rate at 3–4 months follow-up (2)	0	−1^②^	0	0	−1^③^	Very low
		Effective rate at 5–6 months follow-up (5)	0	−1^②^	0	−1^①^	−1^③^	Very low
		Effective rate of 0–1 months follow-up (2)	0	−1^②^	0	−1^①^	−1^③^	Very low
Zhao (2016)	Acupuncture vs western medicine	Effective rate (8)	0	−1^②^	0	0	0	Moderate
Xu (2018)	Acupuncture vs western medicine	Effective rate (6)	0	0	0	−1^①^	0	Moderate

^①^The optimal information size was not enough. ^②^I^2^ value of the combined results was large, and/or confidence intervals overlapped difference. ^③^Suspicion of publishing bias.

## Supplementary Materials

Supplementary Materials S1 Appendix: search strategies. S2 Appendix 2: excluded systematic reviews.
