# Supplementary material for: Effectiveness and Safety of Acupuncture for Migraine: An Overview of Systematic Reviews
Source: Pain Res Manag. 2020 Mar 23;2020:3825617. doi: 10.1155/2020/3825617 (PMC7125485; doi:10.1155/2020/3825617)
Supplement: Supplementary Materials — S1 Appendix: search strategies. S2 Appendix 2: excluded systematic reviews. [file 3825617.f1.pdf]

S1_Appendix Search strategies

MEDLINE via OVID search strategy

1 Headache/28178

2 exp Headache Disorders/35523

3 (headach* or migrain* or cephalgi* or cephalalgi*).mp.112211

4 1 or 2 or 3112275

5 exp acupuncture/1591

6 exp acupuncture therapy/22015

7 exp acupuncture points/5617

8 exp acupuncture ear/366

9 exp auriculotherapy/407

10 exp electroacupuncture/3425

11 exp electric stimulation therapy/78018

12 exp acupressure/649

13 exp moxibustion/1584

14 (acupuncture$ or electroacupuncture$).tw.22047

15 (acupuncture$ or moxibustion).tw.20581

16 5 or 6 or 7 or 8 or 9 or 10 or 11 or 12 or 13 or 14 or 15.101831

17 meta analysis/97965

18 (systematic$ adj review$).tw.129211

19 meta analy$.tw.136494

20 17 or 18 or 19.233209

21 4 and 16 and 20.123

EMBASE search strategy

1 exp "headache and facial pain"/266355

2 (headach* or migrain* or cephalgi* or cephalalgi*).mp.271370

3 1 or 2.289473

4 exp acupuncture/41431

5 exp electroacupuncture/5583

6 exp acupressure/1793

7 exp moxibustion/2365

8 (acupuncture$ or electroacupuncture$).tw.29842

9 (acupuncture$ or moxibustion).tw.28182

10 4 or 5 or 6 or 7 or 8 or 9.43863

11 review.pt.2353253

12 meta analysis/139536

13 (systematic$ adj review$).tw.146726

14 meta analy$.tw.159430

15 11 or 12 or 13 or 14.2531951

16 3 and 10 and 15

S2_Appendix 2 Excluded systematic reviews

References	Reason for exclusion
Coeytaux R R, Befus D. Role of Acupuncture in the Treatment or Prevention of Migraine, Tension-Type Headache, or Chronic Headache Disorders.[J]. Headache, 2016, 56(7):1238-1240.	Not systematic review
Kasle D, Kligler B. Systematic review of acupuncture versus medication for migraine prophylaxis.[J]. Journal of Alternative & Complementary Medicine, 2014, 20(5):42-3.	Commentary
Posadzki P, Albedah A M, Khalil M M, et al. Complementary and alternative medicine for the prevention and treatment of migraine headache: an overview of systematic reviews[J]. Focus on Alternative & Complementary Therapies, 2015, 20(2):58-73.	Not systematic review
Zhang N, Hindiyeh N, Houle T, et al. Systematic Review of Acupuncture vs. Standard Pharmacological Therapy for the Prevention of Migraine[C]//HEADACHE. 111 RIVER ST, HOBOKEN 07030-5774, NJ USA: WILEY-BLACKWELL, 2016, 56: 69-69.	Narrative systematic review
Cheong Y C, Dix S, Hung Y N E, et al. Acupuncture and assisted reproductive technology[J]. Cochrane Database Syst Rev, 2013, 7(7):CD006920.	Not systematic review
Linde K, Allais G, Brinkhaus B, et al. Acupuncture for the prevention of episodic migraine[J]. The Cochrane Library, 2016.	Updated Cochranere view available
Coeytaux R R, Befus D. Role of Acupuncture in the Treatment or Prevention of Migraine, Tension-Type Headache, or Chronic Headache Disorders.[J]. Headache, 2016, 56(7):1238-1240.	Not systematic review

France S, Bown J, Nowosilskyj M, et al. Evidence for the use of dry needling and physiotherapy in the management of cervicogenic or tension-type headache: a systematic review[J]. Cephalalgia An International Journal of Headache, 2014, 34(12):994-1003.	Compared different forms of acupuncture
Linde K, Allais G, Brinkhaus B, et al. Acupuncture for tension-type headache[J]. Cochrane database of systematic reviews (Online), 2009 (1): CD007587.	Updated Cochranere view available
Meissner K, Fässler M, Rücker G, et al. Differential effectiveness of placebo treatments: a systematic review of migraine prophylaxis.[J]. Jama Internal Medicine, 2013, 173(21):1941-1951.	Acupuncture was not the main intervention
Nielsen A. acupuncture for the Prevention of Tension-type Headache (2016)[J]. Explore: The Journal of Science and Healing, 2017, 13(3): 228-231.	Not systematic review
Posadzki P, Albedah A M, Khalil M M, et al. Complementary and alternative medicine for the prevention and treatment of migraine headache: an overview of systematic reviews[J]. Focus on Alternative & Complementary Therapies, 2015, 20(2):58-73.	Not systematic review
Lei Zhao, Yi Guo, Wei Wang, et al. Systematic review on randomized controlled clinical trials of acupuncture therapy for neurovascular headache.[J]. Chinese Journal of Integrative Medicine, 2011, 17(8):580-586.	Narrative systematic review
Hui Z, Min C, Huang D, et al. Interventions for migraine prophylaxis: protocol of an umbrella systematic review and network meta-analysis[J]. Bmj Open, 2015, 5(5):e007594.	Not systematic review
Melchart D, Linde K, Fischer P, et al. Acupuncture for idiopathic headache[J]. Cochrane Database Syst Rev, 2001, 1(1):CD001218.	Updated Cochranere view available
Baeumler P I, Fleckenstein J, Takayama S, et al. Effects of Acupuncture on Sensory Perception: A Systematic Review and Meta-Analysis[J]. Plos One, 2015, 58(2):29-30.	Migraine was not the main research object
Chiang C C, Schwedt T J, Wang S J, et al. Treatment of medication-overuse headache: A systematic review.[J]. Cephalalgia, 2015, 36(4).	Acupuncture was not the main intervention

de Groot F M, Voogt-Bode A, Passchier J, et al. Headache: the placebo effects in the control groups in randomized clinical trials; an analysis of systematic reviews[J]. Journal of Manipulative & Physiological Therapeutics, 2011, 34(5):297-305.	Acupuncture was not the main intervention
Dincer F, Linde K. Sham interventions in randomized clinical trials of acupuncture--a review.[J]. Complementary Therapies in Medicine, 2003, 11(4):235-242.	Migraine was not the main research object
Ernst E. Serious adverse effects after acupuncture - A systematic review (2000-2009)[J]. Perfusion, 2010, 23(6):214-225.	Migraine was not the main research object
Vickers A J, Linde K. Acupuncture for chronic pain[J]. Jama, 2014, 311(9): 955-956.	Not systematic review
Spoerel W E. Acupuncture in chronic pain[J]. The American journal of Chinese medicine, 1976, 4(03): 267-279.	Not systematic review
Fishbain D A. Non-surgical chronic pain treatment outcome: a review[J]. International Review of Psychiatry, 2009, 12(2):170-180.	Not systematic review
Gladstone J P, Dodick D W. Current and emerging treatment options for migraine and other primary headache disorders[J]. Expert Review of Neurotherapeutics, 2003, 3(6):845.	Not systematic review
Peikert A, Wilimzig C, Köhne-Volland R. Prophylaxis of migraine with oral magnesium: results from a prospective, multi-center, placebo-controlled and double-blind randomized study[J]. Cephalalgia, 1996, 16(4): 257-263.	Not systematic review
Facchinetti F, Sances G, Borella P, et al. Magnesium prophylaxis of menstrual migraine: effects on intracellular magnesium[J]. Headache: The Journal of Head and Face Pain, 1991, 31(5): 298-301.	Acupuncture was not the main intervention
Hämäläinen M L. Migraine in children and adolescents: a guide to drug treatment[J]. Cns Drugs, 2006, 20(20):813-820.	Not systematic review
Hao X A, Xue C C, Dong L, et al. Factors associated with conflicting findings on acupuncture for tension-type headache: qualitative and quantitative analyses.[J]. J Altern Complement Med, 2013, 19(4):285-297.	Acupuncture was not the main intervention

Jena M, Mishra S, Pradhan S, et al. Chronic pain, its management and psychological issues: A review[J]. Asian Journal of Pharmaceutical & Clinical Research, 2015, 8(5):42-47.	Acupuncture was not the main intervention
Jindal V, Ge A, Mansky P J. Safety and efficacy of acupuncture in children: a review of the evidence.[J]. J Pediatr Hematol Oncol, 2008, 30(6):431-442.	Migraine was not the main research object
Kreitschmann-Andermahr I, Siegel S, Carneiro R W, et al. Headache and pituitary disease: a systematic review[J]. Clinical Endocrinology, 2013, 79(6):760-769.	Migraine was not the main research object
Malone M D, Strube M J. Meta-analysis of non-medical treatments for chronic pain[J]. Pain, 1988, 34(3): 231-244.	Acupuncture was not the main intervention
Lenssinck M L, Damen L, Verhagen A P, et al. The effectiveness of physiotherapy and manipulation in patients with tension-type headache: a systematic review.[J]. Pain, 2004, 112(3):381-388.	Acupuncture was not the main intervention
Melchart D, Linde K, Fischer P, et al. Acupuncture for recurrent headaches: a systematic review of randomized controlled trials.[J]. Cephalalgia, 2010, 19(9):779-786.	No separate data of acupuncture for migraine
Liu H, Li H, Xu M, et al. A systematic review on acupuncture for trigeminal neuralgia[J]. Alternative Therapies in Health & Medicine, 2010, 16(6):30.	No separate data of acupuncture for migraine
Luedtke K, May A. EHMTI-0091. Physiotherapy interventions for headaches: a systematic review and meta-analysis[J]. Journal of Headache & Pain, 2014, 15(Suppl 1):D38-D38.	Acupuncture was not the main intervention
Becker W J. Headache in Primary Care[J]. Cephalalgia, 2010, 21(2):162-162.	Not systematic review
Molsberger A. Commentary on the Cochrane review of acupuncture for tension-type headache.[J]. Explore, 2009, 5(6):356-358.	Commentary
Muñoz-Ortego J, Solans-Domènech M, Carrion C. [Medical indications for acupuncture: Systematic review][J]. Med Clin, 2016, 147(6):250.	Migraine was not the main research object

Maurizio P, Gianluca S, Marco I, et al. Patient outcome in migraine prophylaxis: the role of psychopharmacological agents[J]. Patient Related Outcome Measures, 2010, 1(5):107-118.	Acupuncture was not the main intervention
Rea P A, Kamani T, Narasimhan M. A review of the management of migraine associated vertigo[J]. Otorhinolaryngologist, 2012, 5(2):80-85.	Not systematic review
Sherman K J, Coeytaux R R. Acupuncture for the treatment of common pain conditions: Chronic back pain, osteoarthritis, and headache[J]. Journal of Clinical Outcomes Management Jcom, 2009, 16(5):224-230.	No separate data of acupuncture for migraine
Vickers A, Cronin A, Maschino A, et al. OA03.01. Acupuncture for chronic pain: an individual patient data meta-analysis of randomized trials.[J]. Bmc Complementary & Alternative Medicine, 2012, 12(1):1-1.	Migraine was not the main research object
Wang D, Yang W, Liu M. Acupuncture for neurological disorders in the Cochrane reviews Characteristics of included reviews and studies[J]. Neural Regeneration Research, 2011, 06(6):440-443.	Migraine was not the main research object
Scott S W, Deare J C, Ferrigno P, et al. Acupuncture for Migraine: A Systematic Review[J]. Australian Journal of Acupuncture & Chinese Medicine, 2006, 1(1).	Insufficient data
D.P. O'Mathúna. Systematic reviews reveal benefits of acupuncture for prevention of migraines and tension-type headaches[J]. Alternative Medicine Alert, 2009, 12(8):94-96.	Not systematic review
Du R, Wang Y, Liu X, et al. Acupuncture for acute migraine attacks in adults: a systematic review protocol[J]. Bmj Open, 2015, 5(4):e006968.	Not systematic review
Coeytaux R R, Befus D. Role of Acupuncture in the Treatment or Prevention of Migraine, Tension-Type Headache, or Chronic Headache Disorders.[J]. Headache, 2016, 56(7):1238-1240.	Not systematic review
Davis M A, Kononowech R W, Rolin S A, et al. Acupuncture for Tension-Type Headache: A Meta-Analysis of Randomized, Controlled Trials[J]. Journal of Pain, 2008, 9(8):667-677.	Not systematic review
Lisa A, Maione L, Vinci V, et al. A Systematic Review of Peripheral Nerve Interventional Treatments for Chronic Headaches.[J]. Annals of Plastic Surgery, 2014, 72(4):439.	No separate data of acupuncture for migraine
